# Ethics of Psychedelic-Assisted Psychotherapy

**DOI:** 10.7759/cureus.102251

**Published:** 2026-01-25

**Authors:** Hana Abbasian

**Affiliations:** 1 Center for Bioethics, Harvard Medical School, Boston, USA

**Keywords:** bioethics framework, healing with psychedelics, medical ethics principles, mental health services, psychotherapy services

## Abstract

Psychedelic-assisted psychotherapy is gaining recognition as a novel approach in mental health care, with the potential to support emotional processing, self-understanding, and personal growth. Early evidence suggests that psychedelic-assisted therapies can catalyze transformative experiences that traditional interventions may not access. This article explores the ethical considerations of these therapies through narrative ethics, which centers the patient’s lived experience and cultural context, and virtue ethics, which emphasizes the clinician’s moral character and relational presence. By integrating these frameworks, this article discusses the importance of holistic and patient-centered care that attends to psychological, social, and cultural dimensions of recovery. Ethical practice in this emerging field involves building trust and inclusivity, ensuring that psychedelic-assisted interventions contribute to resilience, empowerment, and well-being for diverse communities. This discussion aims to provide guidance for clinicians, researchers, and policymakers seeking to navigate the ethical complexities of integrating psychedelics into mental health care.

## Editorial

Psychedelic-assisted psychotherapy is emerging as a transformative approach in mental health care, offering promising interventions for different psychiatric conditions [1]. These therapies can provide experiences of self-reflection, emotional processing, and reconnection to meaning [2]. The resurgence of interest in psychedelics raises questions about the ethical responsibilities of clinicians, the moral dimensions of therapeutic relationships, and the social implications of access to these interventions. The ethics of psychedelic-assisted psychotherapy are analyzed using two complementary frameworks: virtue ethics (focusing on the clinician’s moral character and ethical dispositions) and narrative ethics (centering the patient’s lived story, context, and experiential reality in moral decision-making) [3,4]. Using these frameworks shows how ethical practice in psychedelic therapy requires attentiveness to both relational dynamics and the moral qualities of those providing care.

Narrative ethics and analysis of psychedelic-assisted therapy

Narrative ethics frames ethical practice as relational, context-sensitive, and rooted in the patient’s story [3]. Psychedelic-assisted therapy can frequently evoke emotionally intense and existential experiences, revealing vulnerabilities, traumas, and sources of meaning that traditional talk therapy may not access [1,2]. Clinicians should exercise moral attentiveness (awareness of ethical dimensions inherent in patient interactions) and moral imagination (the capacity to envision and appreciate the patient’s lived experience in its full complexity) [1]. In practice, this means listening deeply to patients, validating their experiences, and supporting the reconstruction of personal narratives that foster growth and healing. Ethical practice through narrative ethics requires recognition of patients as moral agents whose stories guide clinical judgment and therapeutic interventions.

Furthermore, narrative ethics reminds clinicians of the temporal and cultural dimensions of mental health [3]. Patients’ interpretations of psychedelic experiences are influenced by personal history, cultural background, and social context [1,2]. Therefore, psychedelic-assisted psychotherapy should honor patient dignity and the transformative potential of narrative reconstruction.

Virtue ethics and analysis of psychedelic-assisted therapy

Virtue ethics shifts the ethical focus from rules and outcomes to the moral character of the clinician, emphasizing human flourishing [4]. Psychedelic-assisted therapy involves morally and emotionally charged situations [2], requiring clinicians to embody virtues such as practical wisdom, courage, humility, and empathy [1]. These virtues can guide clinicians in navigating the unpredictability of intense psychedelic experiences, allowing them to respond with discernment. Ethical attention to virtue emphasizes that the clinician’s presence, attentional attunement, and moral discernment are themselves therapeutic, shaping the patient’s experience of safety, trust, and openness [4].

Virtue ethics also recognizes that ethical care involves the moral development of both patient and clinician [4]. In psychedelic-assisted psychotherapy, the clinician’s integrity, sensitivity, and relational virtues are inseparable from the ethical quality of care [1]. Under such a framework, the ethical considerations should extend beyond technical competence to include how clinicians embody moral virtues in interactions that shape therapeutic experiences and mental health outcomes [4].

Implications for holistic mental health and recovery

Integrating narrative and virtue ethics illuminates the holistic nature of mental health and recovery in psychedelic-assisted psychotherapy [1,3,4]. These therapies create opportunities for deep self-exploration, emotional integration, and the reconstruction of personal meaning [2]. Holistic mental health recovery requires clinicians to treat the whole person, including their psychological, emotional, social, and cultural dimensions, while supporting environments that support healing, resilience, and empowerment [5]. Narrative ethics encourages attention to each patient’s lived experience [3], emphasizing that recovery is a personal and context-dependent journey shaped by identity, culture, and social connection [3,5]. Virtue ethics complements this by discussing the clinician’s role in building moral sensitivity, empathy, and relational presence, essential qualities that facilitate trust and transformative engagement [1,4].

Holistic care also requires attention to equity, diversity, and inclusion [5]. Therefore, psychedelic-assisted therapy should reach diverse populations and reflect the values, histories, and needs of the communities served. Recovery is relational, and ethical practice recognizes the interplay of social support, cultural identity, and personal agency in well-being [5]. Psychedelic interventions can become tools for integrated mental health care, where emotional, spiritual, and social dimensions are honored alongside psychological and clinical outcomes.

Conclusion

Psychedelic-assisted psychotherapy is an example of the shift toward holistic and patient-centered models of mental health and recovery. Ethical practice in this space encourages recovery that is multidimensional and promotes resilience, self-understanding, and empowerment. As these therapies expand, integrating ethical vigilance with inclusive and culturally attuned approaches ensures that psychedelic-assisted psychotherapy becomes a catalyst for holistic flourishing, dignity, and well-being across diverse communities.
